# Does nighttime hypertension predict long-term kidney outcomes in patients with IgA nephropathy?

**DOI:** 10.1093/ckj/sfaf085

**Published:** 2025-04-24

**Authors:** Takumi Ikeda, Kotaro Haruhara, Takaya Sasaki, Hirokazu Marumoto, Eisuke Kubo, Nobuo Tsuboi, Takashi Yokoo

**Affiliations:** Division of Nephrology and Hypertension, Department of Internal Medicine, The Jikei University School of Medicine, Tokyo, Japan; Division of Nephrology and Hypertension, Department of Internal Medicine, The Jikei University School of Medicine, Tokyo, Japan; Division of Nephrology and Hypertension, Department of Internal Medicine, The Jikei University School of Medicine, Tokyo, Japan; Division of Nephrology and Hypertension, Department of Internal Medicine, The Jikei University School of Medicine, Tokyo, Japan; Division of Nephrology and Hypertension, Department of Internal Medicine, The Jikei University School of Medicine, Tokyo, Japan; Division of Nephrology and Hypertension, Department of Internal Medicine, The Jikei University School of Medicine, Tokyo, Japan; Division of Nephrology and Hypertension, Department of Internal Medicine, The Jikei University School of Medicine, Tokyo, Japan

To the Editor,

The importance of blood pressure control in improving kidney outcomes in patients with immunoglobulin A nephropathy (IgAN) has long been recognized. However, no study has evaluated the prognostic value of ambulatory blood pressure (ABP) monitoring, the most reliable method of blood pressure assessment in routine clinical practice, in patients with IgAN. Our previous cross-sectional study on IgAN identified links between the findings obtained in ABP monitoring at the biopsy diagnosis, as well as clinicopathological findings [1]. In this study, we performed a longitudinal follow-up study to identify the ABP findings at biopsy that were associated with long-term kidney outcomes in the same cohort of IgAN patients. The detailed methods of the study are described in the Supplementary data, Methods.

This study included 113 patients with biopsy-confirmed IgAN. Both groups of patients classified as having daytime and nighttime hypertension had a higher proportion of patients with established clinical risk factors for IgAN progression, such as reduced estimated glomerular filtration rate (eGFR) and overt proteinuria at biopsy (Supplementary data, Table S1) [2]. During a median follow-up period of 8.5 years, 29 patients (26%) had kidney outcomes defined as a 40% reduction in eGFR or the initiation of kidney replacement therapy. A log-rank test showed no significant difference in kidney outcomes when patients were stratified according to daytime hypertension (Fig. 1A). In contrast, significantly more patients who presented with nighttime hypertension had kidney outcomes than those who did not (Fig. 1B). When patients were stratified by nighttime ABP tertiles, we found no significant associations (Supplementary data, Fig. S1). According to a Cox hazard regression model adjusted for reduced eGFR and overt proteinuria, which are established clinical risk factors for IgAN progression, nighttime hypertension did not show an independent association with kidney outcomes (Supplementary data, Table S2).

**Figure 1:**
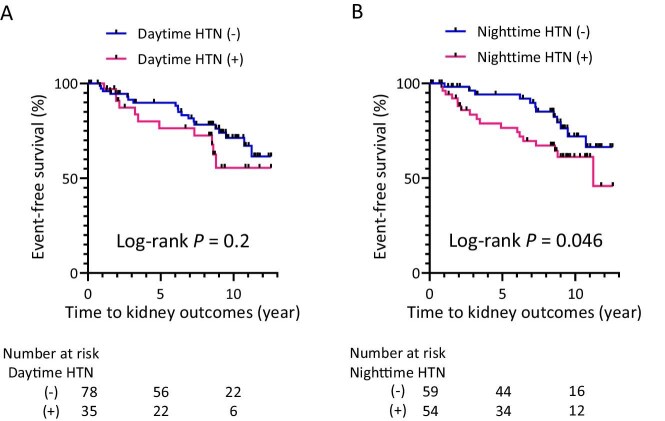
Daytime or nighttime hypertension and kidney outcomes in IgAN patients. (**A**) Kidney survival of the subgroups stratified by daytime hypertension did not differ to a statistically significant extent. (**B**) Kidney survival was significantly worse in patients with nighttime hypertension than in those without hypertension.

The present results are consistent with those of recent studies showing that nighttime ABP is a more accurate predictor of mortality and kidney outcomes than daytime ABP [3, 4]. Several factors may contribute to the predominant association of kidney outcomes in IgAN patients with nighttime hypertension over daytime hypertension, including the compensatory response of the kidneys to reduced sodium excretory capability and the greater reliability of ABP measurements during sleep due to less physical and mental activity [3, 5].

The present results suggest that nighttime hypertension at the time of the biopsy is a clinical finding that may occur secondary to established clinical risk factors for IgAN progression such as a reduced kidney function and overt proteinuria. However, even in situations where these risk factors are known, our present results support the utility of ABP monitoring in IgAN practice for the following reasons. First, unlike chronic kidney dysfunction, which is often irreversible, blood pressure can be modified by treatment. Second, in addition to immunomodulatory therapy for IgAN, antihypertensive therapies with renin–angiotensin–aldosterone system inhibitors and other agents are often necessary to adequately reduce proteinuria. Thus, ABP monitoring is the most reasonable approach, especially for optimizing blood pressure levels to improve long-term kidney outcomes in patients with IgAN. Major limitations of this study were small number of patients and the diversity in the types of antihypertensive medications prescribed, doses and timing, which made it difficult to analyze the effects of these medications on ABP. Determining the effect of therapeutic interventions targeting nighttime hypertension in IgAN patients is an important topic for future research.

In conclusion, this study failed to identify independent relationships between ABP findings at the biopsy, including nighttime hypertension and long-term kidney outcomes, in patients with IgAN. Prospective studies with larger numbers of patients are needed to investigate the involvement of nighttime hypertension in the progression of IgAN.

## Supplementary Material

sfaf085_Supplemental_Files
