# Supplementary material for: Does nighttime hypertension predict long-term kidney outcomes in patients with IgA nephropathy?
Source: Clin Kidney J. 2025 Apr 24;18(5):sfaf085. doi: 10.1093/ckj/sfaf085 (PMC12044330; doi:10.1093/ckj/sfaf085)
Supplement: sfaf085_Supplemental_Files [file sfaf085_supplemental_files.zip › ABP in IgAN_Supplemental_R2_clean.docx]

**Supplemental Materials**

**Does nighttime hypertension predict long-term kidney outcomes**

**in patients with IgA nephropathy?**

Takumi Ikeda, Kotaro Haruhara*, Takaya Sasaki, Hirokazu Marumoto, Eisuke Kubo,

Nobuo Tsuboi*, and Takashi Yokoo

Division of Nephrology and Hypertension, Department of Internal Medicine,

The Jikei University School of Medicine, Tokyo, Japan

**Table of Contents**

Supplemental Methods

Supplemental References

Supplemental Table S1. Patient characteristics at the time of biopsy

Supplemental Table S2. Cox hazard analysis for kidney outcomes

Supplemental Figure S1. Nighttime ABP and kidney outcomes in IgAN patients

**Supplemental Methods**

*Patient selection*

This retrospective study included adult patients (age >18 years) with IgAN who underwent ambulatory blood pressure (ABP) monitoring during admission for a diagnostic kidney biopsy at the Jikei University Hospital, Tokyo, Japan, from April 2010 to March 2014 (1). A kidney biopsy was indicated for patients with an impaired kidney function (estimated glomerular filtration rate [eGFR] <60 mL/min/1.73 m^2^) and/or persistent proteinuria with or without gross or microscopic hematuria. The diagnosis of IgAN was based on typical histopathological features of mesangial proliferative glomerulonephritis, the presence of dominant or co-dominant glomerular IgA deposition detected by immunohistochemistry or immunofluorescence, and the presence of electron-dense mesangial deposits detected by electron microscopy. Patients with other systemic diseases associated with glomerular IgA deposition, including IgA vasculitis, liver cirrhosis, and systemic lupus erythematosus, were excluded. All patients included in this study provided their written informed consent for a kidney biopsy. They were informed that they could withdraw permission for the use of their data at any time. The study protocol was approved by the ethics review board of Jikei University School of Medicine [no. 35-185 (11814)] and conducted in accordance with the Declaration of Helsinki.

*Definitions*

Body mass index (BMI) was calculated using the following equation: BMI (kg/m^2^) = weight (kg) / height^2^ (m). eGFR was calculated from serum creatinine using a modified equation based on Japanese individuals: eGFR = 194 × age^–0.287^ × (serum creatinine) ^–1.094^ (× 0.739 if female) (2). Urinary protein excretion (UPE) was measured using 24-hour urine collection. ABP monitoring was performed 4 to 7 days after admission using a TM-2431C device (A&D, Tokyo, Japan). The ABP parameters were defined as follows: (i) daytime hypertension defined as daytime averaged systolic blood pressure >135 mmHg and/or diastolic blood pressure >85 mmHg, (ii) nighttime hypertension defined as nighttime averaged systolic blood pressure >120 mmHg and/or diastolic blood pressure >70 mmHg, (iii) 24-hour hypertension was defined as the 24-hour averaged systolic blood pressure >130 mmHg and/or diastolic blood pressure >80 mmHg, and (iv) night to day ratio (NDR) was defined as the ratio of the nighttime mean ABP to daytime mean ABP (3). Dipper status was defined as NDR greater than 0.8 and less than 0.9. In the present study, those who did not have dipper status were defined as non-dippers. The salt content of inpatient meals was standardized to 6 g/day. Kidney outcomes were defined as a 40% decline in eGFR at the time of kidney biopsy or the induction of kidney replacement therapy and were observed until June 2023.

*Pathological analysis*

All kidney tissue specimens were obtained via a percutaneous needle biopsy. The tissues were embedded in paraffin, cut into 3 µm sections, and stained with hematoxylin-eosin, periodic acid-Schiff, Masson’s trichrome, and periodic acid silver methenamine. All biopsy samples were subjected to immunohistochemistry or immunofluorescence staining for IgG, IgA, IgM, C3, and C1q. The Oxford scores for mesangial hypercellularity (M), endocapillary hypercellularity (E), segmental sclerosis or adhesion (S), interstitial fibrosis and tubular atrophy (T), and crescents (C) were determined as previously described (4).

*Statistical analyses*

Data are presented as the median with interquartile range (IQR). For comparisons between two groups, the Mann-Whitney test or Fisher’s exact test was used. Survival curve analysis was performed with Kaplan-Meier plots and compared using the log-rank test. Cox hazard regression analysis was performed using urinary protein excretion >500 mg/day and eGFR <60 mL/min/1.73m^2^ as explanatory variables, which were repeatedly shown to be strong predictors in the progression of IgA nephropathy. All reported *p* values were two-sided. P values of <0.05 were considered to indicate statistical significance. Data were analyzed using JMP Pro 18.0.1 (SAS Institute Inc., Cary, NC, USA) and PRISM 10.3.1 (GraphPad Software, La Jolla, CA).

**Supplemental References**

1. Haruhara K, Tsuboi N, Koike K, et al.; Ambulatory blood pressure and tubulointerstitial injury in patients with IgA nephropathy. Clin Kidney J 2015; 8(6):716-21.

2. Matsuo S, Imai E, Horio M, et al.; Revised equations for estimated GFR from serum creatinine in Japan. Am J Kidney Dis 2009; 53(6):982-92.

3. Mancia G, Fagard R, Narkiewicz K, et al.; 2013 ESH/ESC guidelines for the management of arterial hypertension: the Task Force for the Management of Arterial Hypertension of the European Society of Hypertension (ESH) and of the European Society of Cardiology (ESC). Eur Heart J 2013; 34(28):2159-219.

4. Trimarchi H, Barratt J, Cattran DC, et al.; Oxford Classification of IgA nephropathy 2016: an update from the IgA Nephropathy Classification Working Group. Kidney Int 2017; 91(5):1014-1021.

**Supplemental Table S1. Patient characteristics at the time of biopsy**

| Variables | All patients (N=113) | Daytime hypertension | | *P-*value | Nighttime hypertension | | *P-*value |
| --- | --- | --- | --- | --- | --- | --- | --- |
|  |  | Absent  (N = 78) | Present  (N = 35) |  | Absent  (N = 59) | Present  (N = 54) |  |
| Clinical findings |  |  |  |  |  |  |  |
| Male, n (%) | 74 (66) | 45 (58) | 29 (83) | 0.009 | 32 (54) | 42 (78) | 0.009 |
| Age, yr | 38 [31－48] | 35 [31－46] | 42 [35－55] | 0.03 | 33 [28－41] | 43 [35－58] | <0.001 |
| Antihypertensive medications, n (%) | 51 (45) | 30 (39) | 21 (60) | 0.03 | 20 (34) | 31 (57) | 0.01 |
| Number of antihypertensive agents, n (%) |  |  |  | 0.07 |  |  | 0.02 |
| 0, n (%) | 62 (55) | 48 (62) | 14 (40) |  | 39 (66) | 23 (43) |  |
| 1, n (%) | 20 (18) | 11 (14) | 9 (26) |  | 8 (14) | 12 (22) |  |
| 2, n (%) | 19 (17) | 11 (14) | 8 (23) |  | 6 (10) | 13 (24) |  |
| 3, n (%) | 12 (11) | 8 (10) | 4 (11) |  | 6 (10) | 6 (11) |  |
| ACEi or ARBs, n (%) | 38 (34) | 26 (33) | 12 (34) | 0.9 | 17 (29) | 21 (39) | 0.3 |
| Mineralocorticoid receptor antagonists | 2 (2) | 2 (3) | 0 (0) | 0.2 | 2 (3) | 0 (0) | 0.1 |
| β blockers | 5 (4) | 4 (5) | 1 (3) | 0.6 | 3 (5) | 2 (4) | 0.7 |
| Calcium channel blockers, n (%) | 27 (24) | 12 (15) | 15 (43) | 0.002 | 5 (8) | 22 (41) | <0.001 |
| Diuretics | 11 (10) | 8 (10) | 3 (9) | 0.8 | 8 (14) | 3 (6) | 0.1 |
| SGLT2 inhibitors | 0 (0) | 0 (0) | 0 (0) | 1.0 | 0 (0) | 0 (0) | 1.0 |
| Prior history of cardiovascular disease* | 2 (2) | 1 (1) | 1 (3) | 0.9 | 1 (2) | 1 (2) | 1.0 |
| eGFR, mL/min/1.73m^2^ | 66.3  [44.9－82.3] | 72.5  [45.6－87.6] | 56.3  [43.8－73.1] | 0.02 | 75.5  [55.8－90.8] | 55.8  [35.5－73.7] | <0.001 |
| eGFR <60 mL/min/1.73m^2^, n (%) | 47 (42) | 28 (36) | 19 (54) | 0.1 | 19 (32) | 28 (52) | 0.02 |
| Urinary protein excretion, mg/day | 757  [378－1325] | 591.5  [344－2180] | 1123  [724－1793] | 0.001 | 560  [303－1182] | 905  [596－1468] | 0.006 |
| Urinary protein excretion >500 mg/day, n (%) | 79 (70) | 48 (62) | 31 (89) | <0.001 | 34 (58) | 45 (83) | 0.003 |
| 24-hour hypertension, n (%) | 46 (41) | 11 (14) | 35 (100) | <0.001 | 3 (5) | 43 (80) | <0.001 |
| Daytime hypertension, n (%) | 35 (31) | 0 (0) | 35 (100) | <0.001 | 2 (3) | 33 (61) | <0.001 |
| Nighttime hypertension, n (%) | 54 (48) | 21 (27) | 33 (94) | <0.001 | 0 (0) | 54 (100) | <0.001 |
| Non-dipper, n (%) | 64 (57) | 44 (56) | 20 (57) | 0.9 | 26 (44) | 38 (70) | 0.005 |
| Histopathological findings, n (%) |  |  |  |  |  |  |  |
| M1 | 36 (32) | 28 (36) | 8 (23) | 0.2 | 21 (36) | 15 (28) | 0.4 |
| E1 | 30 (27) | 22 (28) | 8 (23) | 0.6 | 15 (25) | 15 (28) | 0.8 |
| S1 | 83 (74) | 58 (74) | 25 (71) | 0.7 | 45 (76) | 38 (70) | 0.5 |
| T1/T2 | 38 (34) | 22 (28) | 16 (46) | 0.07 | 13 (22) | 25 (46) | 0.006 |
| C1/C2 | 67 (59) | 47 (60) | 20 (57) | 0.7 | 38 (64) | 29 (54) | 0.5 |

Footnote: Variables are shown as the median [interquartile range] or number (%). *Cardiovascular disease was defined as myocardial infarction, unstable angina, heart failure, and stroke. Abbreviations: ACEi, angiotensin-converting enzyme inhibitors; ARBs, angiotensin II receptor blockers; SGLT2, sodium-glucose cotransporter-2 inhibitors; eGFR, estimated glomerular filtration rate; M1, presence of mesangial hypercellularity; E1, presence of endocapillary hypercellularity; S1, presence of segmental glomerulosclerosis; T1/T2, tubular atrophy and interstitial fibrosis >25%; C1/C2, presence of cellular or fibrocellular crescents.

**Supplemental Table S2. Cox hazard analysis for kidney outcomes**

| Variables | Crude | | Multivariable model | |
| --- | --- | --- | --- | --- |
|  | HR (95% CI) | *P*-value | HR (95% CI) | *P*-value |
| UPE >500 mg/day | 3.01 (1.05－8.67) | 0.04 | 1.61 (0.54－4.85) | 0.4 |
| eGFR <60 mL/min/1.73m^2^ | 9.04 (3.26－27.0) | <0.001 | 7.89 (2.70－23.0) | <0.001 |
| Nighttime hypertension, present | 2.11 (1.00－4.48) | 0.047 | 1.62 (0.75－3.52) | 0.2 |

Abbreviations: CI, confidence interval; eGFR, estimated glomerular filtration rate; HR, hazard ratio; UPE, urinary protein excretion.
