# Supplementary material for: Does nighttime hypertension predict long-term kidney outcomes in patients with IgA nephropathy?
Source: Clin Kidney J. 2025 Apr 24;18(5):sfaf085. doi: 10.1093/ckj/sfaf085 (PMC12044330; doi:10.1093/ckj/sfaf085)
Supplement: sfaf085_Supplemental_Files [file sfaf085_supplemental_files.zip › Supplemental Figure.pptx]

## Slide 1
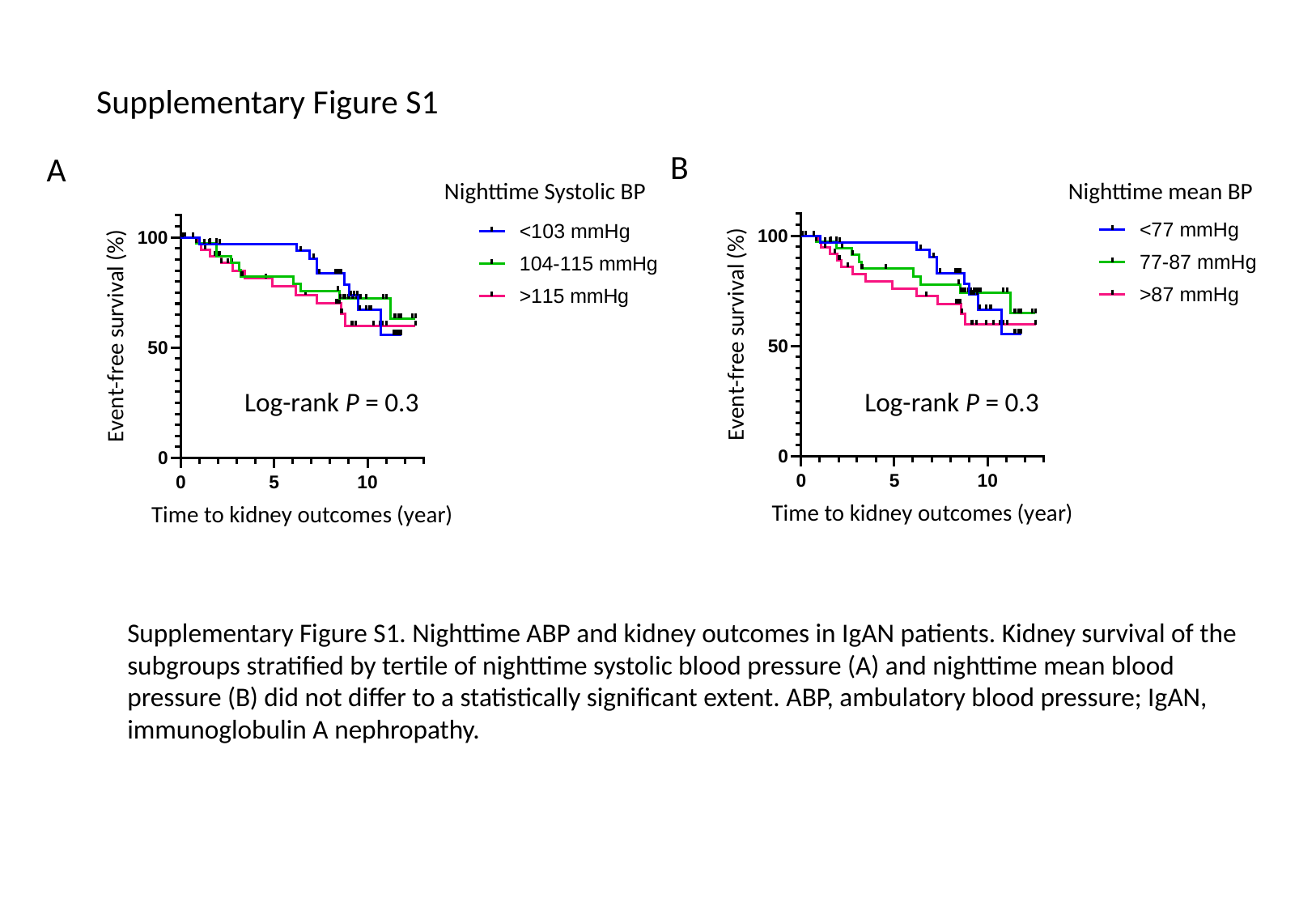

Supplementary Figure S1
B
A
Nighttime Systolic BP
Nighttime mean BP
Log-rank P = 0.3
Log-rank P = 0.3
Supplementary Figure S1. Nighttime ABP and kidney outcomes in IgAN patients. Kidney survival of the subgroups stratified by tertile of nighttime systolic blood pressure (A) and nighttime mean blood pressure (B) did not differ to a statistically significant extent. ABP, ambulatory blood pressure; IgAN, immunoglobulin A nephropathy.
